# Combined airway and esophageal stents implantation for malignant tracheobronchial and esophageal disease: A STROBE-compliant article

**DOI:** 10.1097/MD.0000000000014169

**Published:** 2019-01-18

**Authors:** Yonghua Bi, Jianzhuang Ren, Hongmei Chen, Liangliang Bai, Xinwei Han, Gang Wu

**Affiliations:** a Department of Interventional Radiology, The First Affiliated Hospital of Zhengzhou University; b Department of Ultrasound, Zhengzhou Central Hospital Affiliated to Zhengzhou University, Zhengzhou, China.

**Keywords:** airway, bronchial stents, esophagotracheal fistula, esophagus, fluoroscopy guidance, tracheal stenosis

## Abstract

We aimed to evaluate the safety and efficacy of combined airway and esophageal stents under fluoroscopy guidance and local anesthesia for patients with malignant tracheobronchial and esophageal disease. This retrospective analysis included 35 consecutive patients underwent combined stenting from March 2012 to August 2016. All patients underwent chest computed tomography scans before stenting and during follow-up. Thirty-nine airway stents and 43 esophageal covered stents were implanted. The indication of stenting, technical success and postinterventional complications were collected and analyzed. Thirty-nine airway stents and 43 esophageal covered stents were implanted. Stenting failed in 1 airway stent, and 2 esophageal stents, with technology success rates of 97.4% and 95.3%, respectively. No procedure-related death occurred, only 1 patient died from failure of respiration due to esophagotracheal fistula. The median interval between 2 stenting was 13.0 days. Both dyspnea and dysphasia were significantly relieved after stenting. Restenosis after stenting (7.7%) was the most common complication for airway stenting, all these cases required second stenting. Stent migration (7.0%) was the most common complication after esophageal stenting, 1 case had to receive airway stenting and 1 case received replacement of esophageal stent. During follow up, 23 patients were clinically cured, 2 patients were improved in symptoms, and 1 was invalid. Eight deaths were found in total. The 1-year, 3-year, and 5-year survival rates were 82.4%, 78.8%, and 78.8%, respectively. In conclusion, combined airway and esophageal stents implantation under fluoroscopy guidance and local anesthesia are safe and effective for malignant tracheobronchial and esophageal disease.

## 1 Introduction

Airway and esophagus may be involved simultaneously or successively by esophageal cancer, lung cancer due to the close anatomic relationship.^[1–3]^ Malignant esophagostenosis, tracheostenosis, or esophagotracheal fistula can be caused by these tumors. Those patients are usually poor candidates for surgical treatment due to the advanced tumor stage.^[4–6]^ The palliative treatment simply relieve patient's symptom to improve the patient's quality of life. The self-expandable metallic stent can effectively relieve airway and esophageal stricture, which is widely used clinically for patients with airway^[7–12]^ or esophageal stenosis.^[13–15]^ Patients with advanced lung or esophageal cancer often have respiratory or esophageal distress/fistula caused by tumor invasion, and stenting therapy not only for esophagus but also for airway. Although 80% of esophagotracheal fistula can be palliated by esophageal stenting, an additional seal can also be provided by airway stenting if esophageal stent failed.^[16]^ Small case series have reported combined stents for management of combined malignant airway and esophageal stenosis^[17–19]^ or solely to allow safe placement of the esophageal stent.^[20]^ Combined stenting could be necessary for approximately 9% to 27.5% of patients presenting with tracheobronchial fistula.^[21–23]^ However, majority of these small sample studies were performed under general anesthesia and tracheal intubation^[6,16,19,20,22]^ by using rigid bronchoscopes and esophagoscopes,^[6,16,19,22–24]^ less is known regarding the management of this complex condition under fluoroscopy guidance. In this study, we determined the safety and feasibility of combined stenting in the management of esophagostenosis, tracheostenosis, or esophagotracheal fistula.

## 2 Materials and methods

This retrospective study was approved by the committee board of Zhengzhou University, all procedures were performed in accordance with the guidelines and regulations for clinical study. Informed consents were obtained from all participants enrolled in this study. This study included 35 consecutive patients (25 male, age range: 36–83 years), who underwent combined stenting from March 2012 to August 2016. Combined stents were needed for patients with esophagostenosis combined with tracheostenosis, and airway stent was placed before esophageal stent under this circumstance. Otherwise, the stents were placed according to the order of occurrence of the airway and esophageal stenosis. Esophageal stenting was the first choice for esophagotracheal fistula, and airway stent was used if necessary, such as failure of esophageal stent, recurrence of fistula, or airway stenosis compressed by esophageal stent, and so on. Airway stenting was performed for patients with airway stenosis and/or airway fistula, and esophageal stenting performed if repeated failure or recurrence of airway diseases. Airway stents were intended to remove at a time interval of 3 to 6 months to avoid the long-term complications, especially severe airway stenosis, or left in for the long-term when patients showed a survival time may less than 6 months. Esophageal stents were left in for the long-term, and removal performed only for patients with complications, such as recurrence caused by significant migration, airway compression, or severe restenosis.

### 2.1 Diagnosis and measurement

All patients underwent chest computed tomography (CT) scans before stenting and during follow-up. Pre procedure bronchoscopy/endoscopy performed if necessary, such as confirm diagnosis, treatment for severe stenosis. The airway or esophageal stenosis was diagnosed according to the patient's symptoms, history, and results of chest CT with or without bronchoscopy/endoscopy. The locations, length, and grade of airway and esophageal stenosis was evaluated and measured before stenting. All measurements, proximal and distal landing zones and selection of stent diameter and length were based on chest CT scanning.

### 2.2 Stent types and measurements

**Figure 1:**
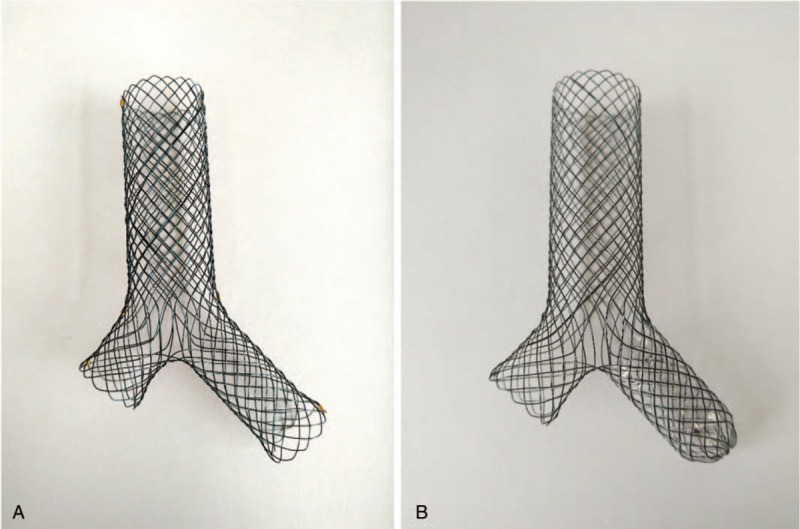
Photos of large Y-shaped airway stent and Y-shaped single-plugged airway stent. Large Y-shaped airway stent was used for patients with stenosis or fistula in bilateral main bronchi. Diameter of the main body, left main bronchus, right main bronchus range 18 to 22 mm, 12 to 14 mm, and 12 to 16 mm, respectively (A); Y-shaped single-plugged airway covered stents were used for left main bronchial stump fistula (B).

All airway covered stents were individually manufactured (Micro-Tech Co Ltd, Nanjing, China). The straight airway covered stent range 18 to 26 mm in diameter and 40 to 100 mm in length. Large Y-shaped stent was used for patients with stenosis or fistula in bilateral main bronchi. Diameter of the main body, left main bronchus, right main bronchus range 18 to 22 mm, 12 to 14 mm, and 12 to 16 mm, respectively, and the length of main body, left main bronchus, right main bronchus were 30 to 60 mm, 15 to 35 mm, and 10 to 25 mm, respectively (Fig. 1). Small y-shaped stent was used for patients with stenosis or fistula in bilateral main bronchi. Diameter of the main body and bronchial limbs range 12 to 18 mm and 10 to 14 mm, and length of main body and bronchial limbs range 15 to 35 mm and 10 to 15 mm, respectively. Two Y-shaped single-plugged airway covered stents were used for left main bronchial stump fistula. All esophageal stents were covered self-expanding stents (Micro-Tech Co Ltd). Of which, 35 stents were tubular esophageal stent; 6 stents were segmented esophagus covered stent, the range of diameter and length was 16 to 18 mm and 100 to 120 mm, respectively. Two bottle shaped esophageal covered stents were used.

### 2.3 Technical details of stenting

**Figure 2:**
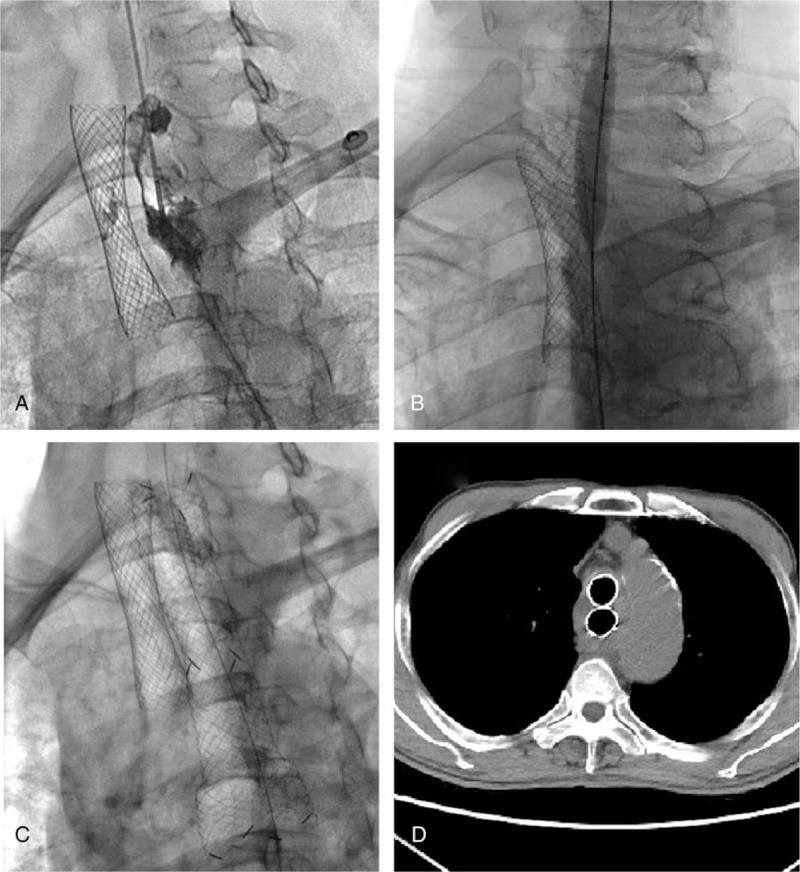
Combined airway and esophageal stents was performed under fluoroscopy guidance for esophagostenosis, tracheostenosis, and esophagotracheal fistula. A sever esophagostenosis and minor esophagotracheal fistula was found after airway stenting (A); The esophagus was predilated with a balloon (B), and then a covered esophagus stent was implanted (C). The double stents were well located with no complication during follow-up (D).

All stent implantation and removal was performed under fluoroscopic guidance with local pharyngeal anesthesia (5 mL of 2% lidocaine aerosol). A 12 to 14 F long sheath was prepared to manage the airway and ventilation during airway stent placement or removal.^[25]^ Balloon dilatation of stent was performed immediately after stenting due to insufficient expansion or before stent implantation if necessary. A 5F vertebral artery catheter (Cook Corporation, Bloomington) was introduced, a 0.035-inch stiff guide wire (Cook Corporation) was then advanced, and stent was implanted along the stiff wire (Fig. 2). If a Y-shaped stent was needed, 2 0.035-inch guide wires were inserted into the bilateral main bronchi, and the stent was sent via the 2 stiff guide wires.^[26]^ For stent removal, a 10 to 14 F long sheath was inserted via a stiff guide wire and then inserted an extractive hook to withdraw stents.

### 2.4 Definition and assessment

Technical success of stenting was defined as exact stenting with no severe procedure-related complications. Peroperative stenting failure was defined as severe stent migration or repeated migration required stent removal; or stent release failure due to the technology factor. The Hugh–Jones classification was used to assay dyspnea before and after airway stenting. Dysphagia was also evaluated before and after esophageal stenting according to previous report^[13]^ (0, normal diet; 1, partial solid food without the nutritional support; 2, semisolid food without the nutritional support; 3, the ability to swallow liquids only; and 4, complete dysphagia with the nutritional support). Clinical cure defined as successful management of disease without complications or symptom.

### 2.5 Follow-up

All patients underwent chest CT within 1 week after stenting to confirm the location of stents. The chest CT, clinical examination, or bronchoscopy/endoscopy were performed 1 month after stenting and then about every 3 months thereafter during follow-up. Telephone follow-up was carried out for patients unable to visit hospital.

### 2.6 Statistical analysis

All statistical calculations were performed using Prism 5.0 software (GraphPad Software Inc, San Diego, CA). Descriptive statistics were used to describe the conditions of patients. Continuous variables are summarized as mean ± SE. The student *t* test was performed to compare continuous variables. Fisher exact test was used for compare incidence of complications. A *P* < .05 was considered statistically significant.

## 3 Results

### 3.1 Technical success

One airway stent failed to release due to the stent and binding wire was wound together. After repeated attempts, the stent had to removed, and airway stent was successfully placed again 1 week later. A chest CT obtained within 1 week after stenting confirmed the correct location of all airway stents. The correct location of esophagus stents within 1 week after stenting was confirmed in 35 patients. Two esophageal stents failed to treat fistula due to migration 2 and 5 days after stenting, and airway stents were used. The technology success rates were 97.4% and 95.3% for airway and esophageal stent, respectively. The mean stenting time was 30.4 ± 3.4 minutes and 34.5 ± 3.4 minutes for airway and esophagus stents, respectively (Table 1). The median interval between the airway and esophageal stents for combined cases was 13.0 days (range, 0–448 days).

**Table 1:**
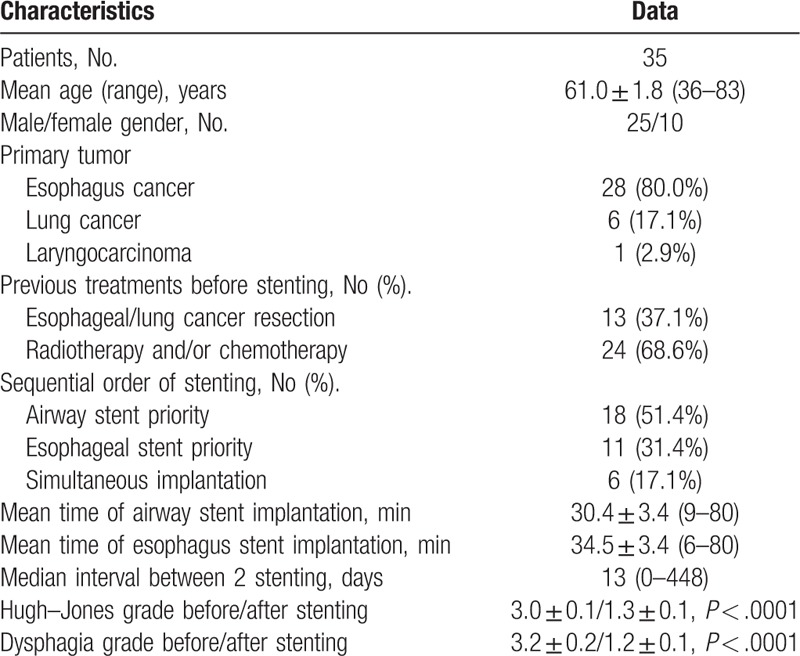
The patients’ characteristics.

### 3.2 Stent implantation

For the sequential order of stenting, as shown in Table 1, 18 (51.4%) patients underwent first airway stents implantation, and 6 (17.1%) patients received simultaneous airway and esophageal stenting. Thirty-nine airway stents and 43 esophageal covered stents were implanted, including 5 esophageal stents for second stenting after removal. Types of stents were shown in Table 2, which included straight airway covered stent (n = 16), large Y type airway stent (n = 17), small y type airway stent (n = 4), and Y-shaped single-plugged airway stent (n = 2); Esophageal stents included tubular esophageal covered stent (n = 35), segmented esophagus covered stent (n = 6), and bottle shaped esophageal covered stent (n = 2).

**Table 2:**
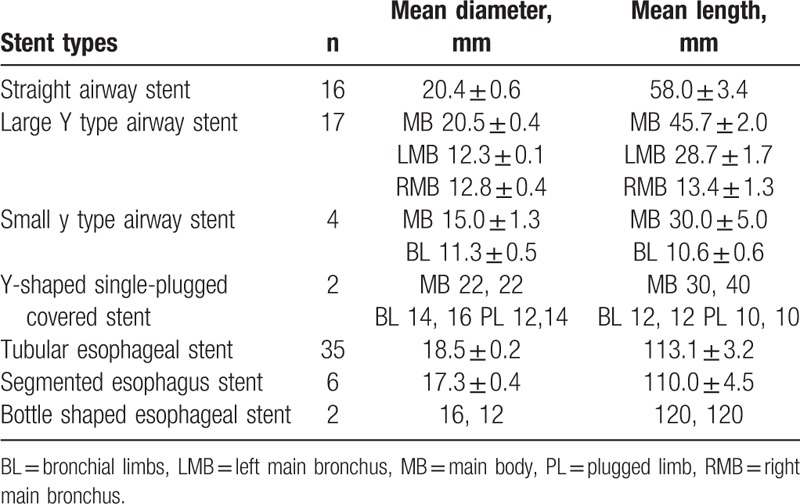
The stent types and dimensions.

### 3.3 Indications for stenting

Esophagotracheal fistula after resection or radiotherapy of esophageal carcinoma (n = 7), airway stent restenosis (n = 3), esophagostenosis combined tracheostenosis due to esophageal carcinoma (n = 3) were the main indications for airway stenting. Recurrence of esophagotracheal fistula after stenting (n = 6), esophagotracheal fistula (n = 3), malignant esophagostenosis (n = 4) were the most common indications for esophageal stenting. Esophagostenosis combined tracheostenosis due to esophageal cancer or lung cancer (n = 12) and esophagotracheal fistula with/without esophagotracheal stenosis (n = 10) were indications for both airway and esophageal stenting (Table 3).

**Table 3:**
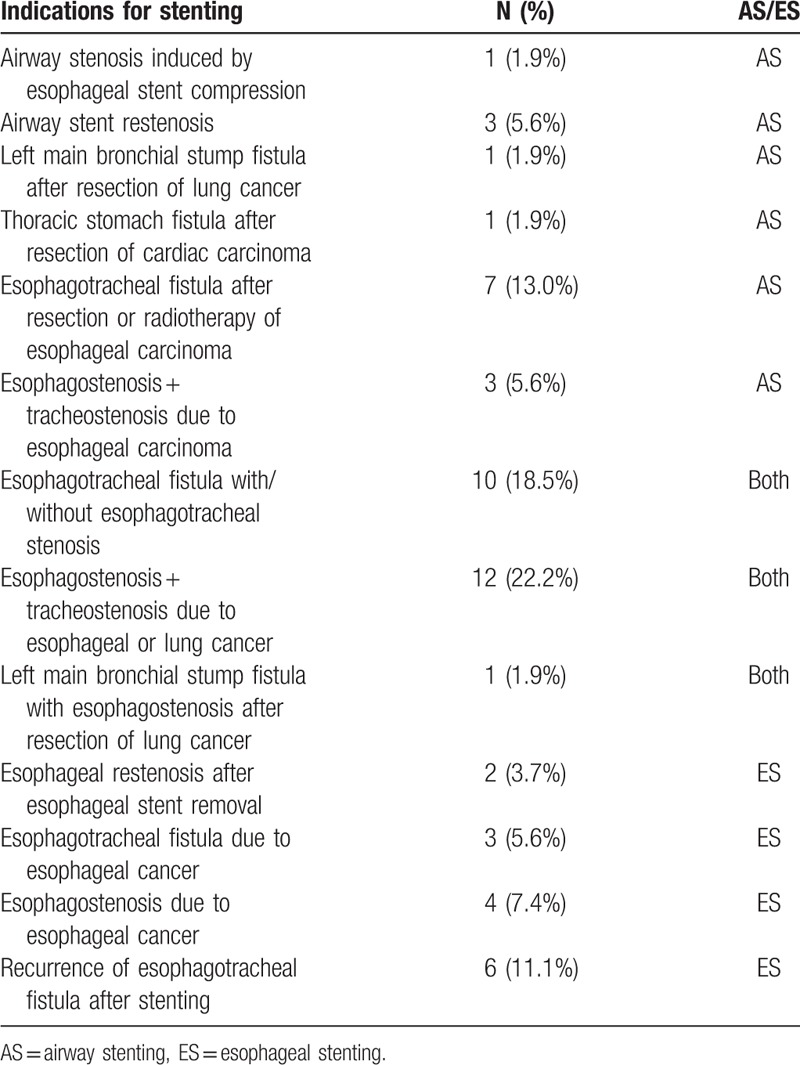
Indications for stenting.

### 3.4 Peroperative complications

**Figure 3:**
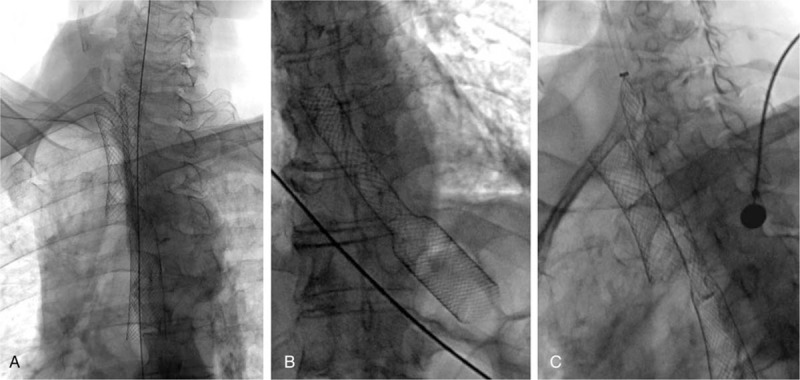
The migrated esophagus stent was relocated under fluoroscopy guidance. Straight airway covered stent and bottle shaped esophageal covered stent were used (A); the esophagus stent migrated about 2 weeks after stenting (B). A long sheath was inserted via the stiff hydrophilic guide wire; the migrated stent was adjusted with its thread (C).

One patient died from failure of respiration 3 days after esophageal stenting for treatment of recurrence esophagotracheal fistula after airway stent removal. Esophageal stenting showed a higher incidence of total complications. Restenosis after stenting (7.7%) was the most common complication for airway stenting, all these cases required second stenting. Stent migration (7.0%) was the most common complication after esophageal stenting (Fig. 3). Airway compression was the main and severe complication after esophageal stenting, which could be found in 3 (7.0%) cases (Table 4). All cases underwent esophageal stent removal, 1 case had to receive airway stenting and 1 case received replacement of esophageal stent.

**Table 4:**
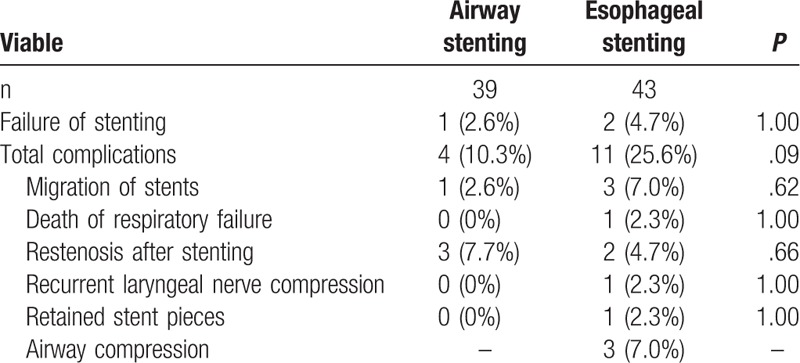
Complications of stenting.

### 3.5 Stent removal

Eleven esophagus stents and 2 airway stents were withdrawn in 11 patients; the mean indwelling duration of stent ranged 2 to 270 days. Two esophagus stents and 1 airway stent were regularly removed according to doctor's advice to avoid long-term complication. Two esophagus stents and 1 airway stent were removed due to repeated migration of the stent. Three esophagus stents were withdrawn due to airway compression, and 2 due to restenosis of esophageal stent. Four esophagus stents and 1 small y type airway stent were implanted immediately after esophagus stent removal owing to failure recovery of esophagotracheal fistula (n = 1), restenosis of esophagus (n = 3), or retained stent pieces (n = 1).

### 3.6 Efficacy assessment

The symptoms of esophagotracheal fistula improved after placement of covered stent. Both respiratory and dysphagia symptoms were immediately improved in all patients after stenting. The mean Hugh–Jones grade decreased from 3.0 ± 0.1 to 1.3 ± 0.1 after airway stenting (*P* < .0001). The mean dysphagia grade decreased from 3.2 ± 0.2 to 1.2 ± 0.1 after esophageal stenting (*P* < .0001). During follow up, 23 patients were clinically cured, 2 patients were improved in symptoms, and 1 was invalid.

### 3.7 Follow-up

**Figure 4:**
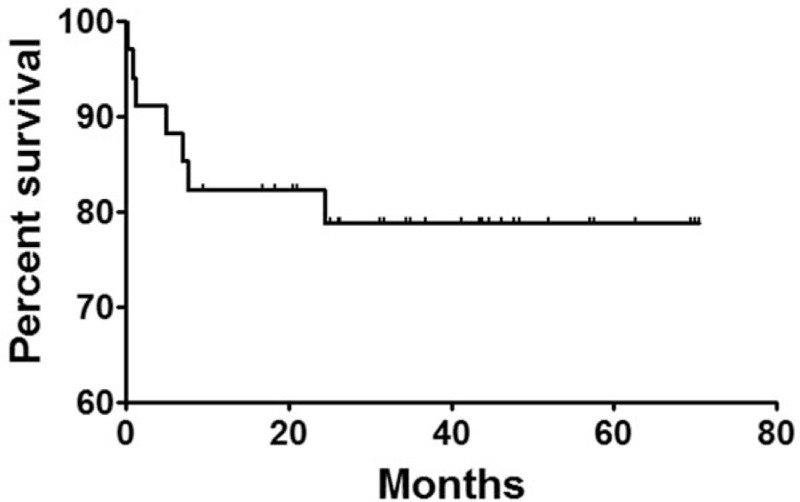
Survival rate follow up. The 1-year, 3-year, and 5-year survival rates were 82.4%, 78.8%, and 78.8%, respectively.

One patient was lost to follow up. The remaining 34 patients were followed up for 19.6 to 70.5 months, with a mean of 40.8 ± 3.1 months. During follow up, 4 patients died of tumor progression, 1 person died each from massive hematemesis, respiratory failure, and severe lung infection. Eight deaths were found in total, including the 1 died 3 days after stenting. The 1-year, 3-year, and 5-year survival rates were 82.4%, 78.8%, and 78.8%, respectively (Fig. 4).

## 4 Discussion

Patients with advanced lung or esophageal cancer often have respiratory or esophageal distress/fistula caused by tumor invasion. It was reported that combined stenting of airways and esophagus in the palliative treatment of patients with lung/esophageal cancer is feasible and efficient, but should be reserved to selected patients with end-stage disease and severe symptoms.^[6]^ The incidence of early life-threatening complications is 20%, including deaths following esophagotracheal fistula and massive hematemesis.^[4–6]^ Compared with self-expandable metallic stents, the silicone stents might more rigid but less reactive to the airway or esophageal wall,^[4,27]^ which were used to prevent a recurrence of esophagus stenosis.^[18]^

Traditionally, stent was placed most commonly through the esophagus, but occasionally through the airway. Covered esophageal stent was used for malignant airway-esophageal fistula. Patients with esophageal cancer might require airway stent for airway complications, such as airway-esophageal fistula,^[1]^ airway compression,^[18]^ and both airway-esophageal fistula and airway compression.^[28]^ Nasir et al^[24]^ have chosen a proactive approach to double stenting patients early, rather than wait until airway complications have observed.

Airway and/or esophageal stent insertion provided an effective approach to improve the quality of life in patients with malignant airway–esophageal fistula.^[23]^ However, only 9% of patients received combined stents under endoscopic guidance. The postoperative morbidity occurred in 28% of patients after combined stents implantation, including pneumonia or respiratory failure, right-side heart failure, and intestinal perforation.^[24]^ Besides, excessive compression between combined stents may cause the esophagotracheal fistula or massive bleeding.^[4]^ In our study, no patients experienced life-threatening complications or procedure-related death after combined stents implantation. The total complication rates were 10.3% and 25.6% for airway and esophageal stents. Our data showed that combined stenting effectively the relieved symptom, both dyspnea and dysphagia were significantly relieved after stenting. Airway compression (7.0%) was the main and severe complication after esophageal stenting in our study, which was lower than the previous report.^[19]^

Combined stenting has also been reported in the treatment of esophagotracheal fistulas using rigid bronchoscopes and esophagoscopes;^[16,22]^ however, less than 10 patients had fistulae before stent therapy.^[6,18]^ Nomori et al reported a high risk of fistula occurring due to necrosis of airway/esophageal walls, and occurrence/recurrence of fistula due to growth of fistula after stenting.^[4]^ Besides, considering that there is a high risk of fistula occurring after combined stents placement in the future, the covered metallic stents should be used in esophageal cancer invading the airway, even for patients without esophagotracheal fistula.^[4]^ In this study, no fistulae were caused by combined stenting.

Although airway stents can be inserted under fluoroscopic guidance,^[11,12,26]^ most of airway stents were often performed under general anesthesia and tracheal intubation.^[6,16,19,20,22]^ However, patients with malignant stenosis and/or fistula are usually poor candidates for surgical treatment due to the advanced tumor stage.^[4–6]^ The esophageal metallic stents can be placed under local anesthesia,^[14,15]^ and inserted under fluoroscopic guidance.^[13]^ All stents were implanted under local anesthesia and fluoroscopic guidance in this study.

This study had some limitations. First, this is a retrospective study in a single institution. Second, the sample size is still small, making it difficult to make definitive conclusions regarding this technique.

In conclusion, combined airway and esophageal stents implantation under fluoroscopy guidance and local anesthesia are safe and effective for malignant tracheobronchial and esophageal disease.

## Author contributions

Study design was done by HXW and WG; Data collection was done by BYH, RJZ, CHM, and BLL; Data analysis was done by BYH, RJZ, CHM, and BLL; Written by BYH, RJZ, and WG; Study was approved by HXW, and WG.

**Conceptualization:** Xinwei Han, Gang Wu.

**Data curation:** Yonghua Bi, Jianzhuang Ren, Hongmei Chen, Liangliang Bai.

**Formal analysis:** Yonghua Bi, Jianzhuang Ren.

**Funding acquisition:** Yonghua Bi.

**Investigation:** Yonghua Bi, Jianzhuang Ren, Hongmei Chen, Liangliang Bai.

**Methodology:** Hongmei Chen.

**Project administration:** Xinwei Han, Gang Wu.

**Resources:** Yonghua Bi.

**Software:** Hongmei Chen, Liangliang Bai.

**Supervision:** Xinwei Han, Gang Wu.

**Validation:** Xinwei Han, Gang Wu.

**Visualization:** Xinwei Han.

**Writing – original draft:** Yonghua Bi, Jianzhuang Ren, Hongmei Chen.

**Writing – review and editing:** Xinwei Han, Gang Wu.
